# Retraction: miR-211 suppresses hepatocellular carcinoma by downregulating SATB2

**DOI:** 10.18632/oncotarget.28837

**Published:** 2026-04-24

**Authors:** Guixing Jiang, Yunfu Cui, Xin Yu, Zhengrong Wu, Guoping Ding, Liping Cao

**Affiliations:** ^1^Department of Hepatopancreatobiliary Surgery, Sir Run Run Shaw Hospital, School of Medicine, Zhejiang University, Hangzhou, China; ^2^Department of Hepatopancreatobiliary Surgery, Second Affiliated Hospital of Harbin Medical University, Harbin, China; ^3^Department of Biochemistry and Molecular Biology, Institute of Basic Medical Sciences, Chinese Academy of Medical Sciences, Beijing, China

**This article has been retracted:** Oncotarget has completed its investigation of this paper. The review identified several instances of external image duplication and overlap:

- Figure 4D: The GAPDH blot is a duplicate of blot in Figure 4D of an earlier published unrelated paper [1].
- Figure 3B: The Transwell assay images were found in Figures 5A and 7C of earlier submitted and already retracted article [2].
- Figure 5A: The GAPDH blot was duplicated in Figure 4B of a retracted article [3].
- Figure 7E: The GAPDH blot was found in Figure 4A of article [4] and Figure 5B of article [5], which were submitted before the Oncotarget paper, as well as in Figure 4A of [6], Figure 5B of [7], and Figure 4D of [8].

Additionally, the cell lines SMMC-7721 and Hep-G2, used in this study as hepatocellular carcinoma cell lines, are known to be problematic [9–11]. These issues, combined with the identified duplications, raise significant concerns regarding the reliability of the data and conclusions. Consequently, the Editorial decision has been made to retract this paper. The authors were notified of this decision.

Original article: Oncotarget. 2015; 6:9457–9466. 9457-9466. https://doi.org/10.18632/oncotarget.3265
